# On the Neurophysiological Mechanisms Underlying the Adaptability to Varying Cognitive Control Demands

**DOI:** 10.3389/fnhum.2018.00411

**Published:** 2018-10-16

**Authors:** Nicolas Zink, Ann-Kathrin Stock, Amirali Vahid, Christian Beste

**Affiliations:** Cognitive Neurophysiology, Department of Child and Adolescent Psychiatry, Faculty of Medicine, Technische Universität Dresden, Dresden, Germany

**Keywords:** cognitive control, EEG, working memory load, machine learning, predictive feature

## Abstract

Cognitive control processes are advantageous when routines would not lead to the desired outcome, but this can be ill-advised when automated behavior is advantageous. The aim of this study was to identify neural dynamics related to the ability to adapt to different cognitive control demands – a process that has been referred to as ‘metacontrol.’ A sample of *N* = 227 healthy subjects that was split in a ‘high’ and ‘low adaptability’ group based on the behavioral performance in a task with varying control demands. To examine the neurophysiological mechanisms, we combined event-related potential (ERP) recordings with source localization and machine learning approaches. The results show that individuals who are better at strategically adapting to different cognitive control demands benefit from automatizing their response processes in situations where little cognitive control is needed. On a neurophysiological level, neither perceptual/attentional selection processes nor conflict monitoring processes paralleled the behavioral data, although the latter showed a descriptive trend. Behavioral differences in metacontrol abilities were only significantly mirrored by the modulation of response-locked P3 amplitudes, which were accompanied by activation differences in insula (BA13) and middle frontal gyrus (BA9). The machine learning result corroborated this by identifying a predictive/classification feature near the peak of the response-locked P3, which arose from the anterior cingulate cortex (BA24; BA33). In short, we found that metacontrol is associated to the ability to manage response selection processes, especially the ability to effectively downregulate cognitive control under low cognitive control requirements, rather than the ability to upregulate cognitive control.

## Introduction

Cognitive control is one of the key competencies required to adapt to dynamic environments and to control our behavior (Botvinick et al., 2004; Gruber and Goschke, 2004; Bocanegra and Hommel, 2014; Larson et al., 2014). While cognitive top–down control processes are advantageous when automated behavior (routines) would not lead to the desired outcome (Miller, 2000; Miller and Cohen, 2001; Botvinick et al., 2004; Ochsner and Gross, 2005; Botvinick and Braver, 2015),exerting cognitive control in situations where automated behavior is beneficial can have detrimental effects on behavior (Olivers and Nieuwenhuis, 2005; Taatgen et al., 2009; Bocanegra and Hommel, 2014; Stock et al., 2016c). Individuals who are able to flexibly adapt to situations where less cognitive control is required might benefit more from rather automated behavior, which is faster and less capacity demanding. Demands on cognitive control can vary as a function of many factors, including the complexity of a given task rule (Rubinstein et al., 2001; Pieczykolan and Huestegge, 2017): If the task rule is more/less complex, demands on cognitive control are higher/lower. Lower cognitive controls demands are associated with more automatic response processes, which tend to be less error prone than cognitive control mechanisms, once they are in place (Möschl et al., 2017).

Against this background, it has increasingly been recognized that cognitive control processes sub-serving goal-directed actions require a dynamic, context sensitive balancing of how much cognitive control is ‘invested’ (Goschke and Bolte, 2014; Hommel and Wiers, 2017): Behavior would be ill-advised by a system that pursues goals irrespective of what is ‘optimal’ in a given situation (Hommel and Wiers, 2017). It would be much more beneficial and expedient to adjust cognitive control or response selection processes depending on task rule complexity because both of those processes are known to be capacity-limited (Meyer and Kieras, 1997; Engle and Kane, 2003; Kane and Engle, 2003; Tombu and Jolicoeur, 2003; Lavie et al., 2004; Marois and Ivanoff, 2005; Sigman and Dehaene, 2008; Engle, 2010; Lavie, 2010; Yildiz et al., 2013; Yildiz and Beste, 2015). In this context, the central question is *how* information is processed and responses are selected (Doya, 2008; Goschke and Bolte, 2014) in situations with different control requirements. This aspect has been referred to as ‘metacontrol’ (Goschke and Bolte, 2014; Hommel and Wiers, 2017). Several models were proposed that describe the ability to adapt to different aspects of cognitive control. In a model by Shenhav et al. (2013) emphasis was put on the role of an internal evaluation of the expected value of control (EVC; Shenhav et al., 2013), which would then result in up- or downregulation of cognitive control capacities. Others have described how individuals might strategically adapt to errors (Ullsperger et al., 2014) or conflicts (Reuss et al., 2014). Yet, the neurophysiological mechanisms related to the ability to strategically adapt to different cognitive control demands, which vary as a function of task rule complexity have remained elusive^1^.

To examine this question, we used a paradigm that consists of two complementary tasks with high and low cognitive control demand (Bocanegra and Hommel, 2014). Based on the aforementioned findings, we assumed that individuals who can flexibly adjust to varying demands and effectively tone down their top–down control mechanisms when control requirements are low, should perform better. This effect should be most pronounced in the easy task, where automated behavior is beneficial. Yet still, such an improvement should always be related to the performance in the hard task in order to account for inter-individual performance differences. We therefore calculated a behavioral score that accounts for the relation of speed and accuracy and the performance difference between the two tasks. Those who displayed larger task differences due to better task performance in the easy task were classified as the “high adaptability group,” while those with smaller task differences were termed “low adaptability group” (please see “Materials and Methods” and “Results”).

In order to identify the neurophysiological processes and functional neuroanatomical networks that are differently modulated by varying cognitive control demands, we recorded an EEG. The advantage of electrophysiological (EEG) techniques and event-related potentials (ERPs) in this context is that they allow to dissociate different cognitive sub-processes involved in information processing on the basis of their temporal occurrence in a high temporal resolution. When combined with source localization techniques, it is possible to identify brain regions being associated with above-mentioned ‘metacontrol’ dynamics; i.e., *how* individuals differently adapt to varying cognitive control demands. Regarding neurophysiological processes that may be modulated by the adaptability to different cognitive control demands, ERPs like the mid-central N2 and N450 (Larson et al., 2014) may be important to consider. Both have been shown to be enhanced in case of larger cognitive control demands (Botvinick et al., 2004; Folstein and Van Petten, 2008; Larson et al., 2014; Stock et al., 2016c). Although the difference between those ERPs is not entirely clear, the N2 has repeatedly been associated with conflict adaptation and monitoring as well as cognitive effort (Botvinick et al., 2004; Folstein and Van Petten, 2008; Larson et al., 2014; Chmielewski et al., 2016) and the N450 has been shown to reflect detection, monitoring, and resolution of conflicts (Szûcs and Soltész, 2012; Vanderhasselt et al., 2012; Larson et al., 2014). The finding that the N450 has previously shown larger task/control demand-induced differences in a comparable experimental paradigm than the N2 (Stock et al., 2016d; Zink et al., 2018), suggests that we might find the N450 to better reflect effects of task adaptation than the N2. Specifically, we would expect smaller (i.e., less negative) N450 amplitudes during the low control demand task in individuals with a high adaptability (i.e., metacontrol abilities) than in individuals with low adaptability. The dual mechanism framework of cognitive control (Braver, 2012) has been shown to give rise to two different families of P3-like ERP potentials (Barcelo et al., 2002; Barceló and Cooper, 2017), with one P3 originating from stimulus-driven frontal attention mechanisms during task processing, and another originating from temporal–parietal activity associated with attention and appears related to subsequent memory processing (Polich, 2007). In this study, the adaptation to varying cognitive control demands may also be reflected by modulations of the parietal P3 component, which is known to represent the process of mapping a stimulus onto an appropriate response (Verleger et al., 2005; Twomey et al., 2015; Verleger et al., 2015). In a previous study using a comparable experimental paradigm, we found the response-locked parietal P3 to also reflect differences in cognitive control requirements (Stock et al., 2016d) and observed larger amplitudes in case of low than in case of high control demands. In dual task situations (Polich, 2007) or during processes of early vision (Schubö et al., 2001), this amplitude increase has repeatedly been suggested to reflect the attentional resources left over by the primary task. Based on the assumption that cognitive control is not only more effortful, but also demands more attentional resources than a rather automatic approach to response selection, we expected larger response-locked parietal P3 amplitudes during the low control task in individuals with a high adaptability (i.e., metacontrol abilities) than in individuals with low adaptability. The reasoning behind this hypothesis is that if individuals with higher metacontrol are better able to refrain from engaging top-down strategies in situations with low control requirements, this will leave more attentional resources at the disposal of the participant, which should be reflected by a larger parietal P3 component. Consequentially, task differences in parietal P3 amplitude should be larger in individuals who can flexibly adapt their response selection procedure to a more automatic or a more controlled mode (i.e., in the high adaptability group) compared to those who lack this flexibility (low adaptability group). However, as stimulus-driven frontal attention mechanisms reflected by a fronto-central P3 modulation may also account for differences in the adaptability toward different levels of cognitive control, the fronto-central P3 was also assessed. Moreover, as larger P3 amplitudes are thought to reflect changes in the availability of attentional resources, we also quantified attentional processes as reflected by the P1 and N1 ERP components (Luck et al., 2000; Herrmann and Knight, 2001). In line with our previous paper, we expected to find larger amplitudes in case of low control demands. But given that both ERPs represent early attentional processing of incoming stimuli (Luck et al., 2000; Schneider et al., 2012) and occur much earlier than the P3, we are skeptical whether the attentional resources available at the time point of stimulus-response mapping could already show in those earlier processes. Hence, we did not expect to find group differences in those two ERPs.

In addition to those classical ERP analyses, we chose to complement our methodological approach with a machine learning approach using support vector machines (SVMs) (Boehler et al., 2008; Plewan et al., 2016; Stock et al., 2016b). Importantly, this SVM approach is unbiased by prior findings/hypotheses and allows to identify neurophysiological features from the entire time series (and not just the ERP peaks) that best classify (or predict, to use the common machine learning term) behavioral performance. The main reason for choosing this approach is that the selection and analysis of ERPs is usually based on previous correlative findings and furthermore limited to minima, maxima, or pre-defined time windows in the course of the neurophysiological times series. This excludes a wealth of data “in between” the investigated ERPs. This is problematic as neurophysiological signals are usually composed of different signals which may vary in latency and thus not all be (best) reflected by composite ERP peaks. Moreover, it has recently been pointed out (Bridwell et al., 2018) that component peaks may detract from the ability to detect EEG features that relate to behavior. Further, components are often defined by latent cognitive constructs that may not necessarily be a good characterization of neural computations. In fact it has been shown that transient activity is important to consider for the dynamics of cognitive functions (Bridwell et al., 2018). Since theoretical concepts dealing with the question how to strategically adapt to different cognitive control demands stress the dynamics of cognitive processes (Goschke and Bolte, 2014; Hommel and Wiers, 2017), the ability to be able to detect transient aspects in EEG activity (i.e., processes not necessarily captured by ERP-peaks) machine learning approaches are particularly suitable. Moreover, the SVM approach enables us to overcome those issues and consider the entire time series to identify the neurophysiological features and brain activation differences which best differentiate between the high and low adaptability groups.

Regarding the functional neuroanatomical structures that are associated with metacontrol (i.e., how efficiently individuals can adapt to different control requirements), the prefrontal cortex is of importance, as it plays a key role in cognitive control (Miller and Cohen, 2001; Stuss and Knight, 2002) and has been shown to be associated with the adaptation of response strategies toward statistically optimal behavior (Koechlin, 2016). Especially the ACC seems to have a monitoring function that serves to regulate both cognitive and emotional processing (Bush et al., 2000), conflict detection (van Veen et al., 2001; Botvinick et al., 2004; Carter and van Veen, 2007), control execution (Ridderinkhof et al., 2004; Nee et al., 2007; Shackman et al., 2011; Niendam et al., 2012), and adaptive behaviors (Holroyd and Coles, 2002). It was argued that the underlying function of the ACC is to raise the threshold for initiating ‘wanted’ behavior, while the threshold is lowered for all “unwanted” alternatives (Paus, 2001) or “energizing” the neural systems that needed to make the decisions and initiate the responses (Stuss et al., 2005). While these theories assume that activation in this region is mostly useful for boosting weak processes in order to establish top down control, the complementary idea is that the ACC is involved in metacontrol, or the modulation between upregulating processes (i.e., exerting top–down control) and downregulating processes/keep the ongoing processes unaltered (automatic mode). Shenhav et al. (2013) proposed that the diversity of functions associated with the ACC can be understood in terms of a single underlying function, the allocation of control based on the evaluation of the EVC. Compared to individuals from the low adaptability group, individuals from the high adaptability group might be better in assigning the optimal amount of cognitive control that needs to be invested. Therefore, we expected that the hypothesized differential modulation of neurophysiological processes between the low and high adaptability groups should be related to activation differences within the ACC.

## Materials and Methods

### Sample

For this study, *n* = 227 healthy young participants (165♀, 62♂) aged 18–32 (mean 23.7, SD 3.1) were tested. As gender is clearly unbalanced in favor of female participants, potential impacts on the generalizability of the results cannot be ruled out. None of the included participants reported neurologic or psychiatric disorders and all participants stated to have normal or corrected-to-normal vision. Based on Beck Depression Inventory (BDI) scores (Beck et al., 1961), depression was ruled out (mean score 4.69, SD 4.34). All participants gave written informed consent and received a reimbursement of 25 €. The study was approved by the ethics committee of the TU Dresden and participants were treated in accordance with the declaration of Helsinki.

### Task

A modified version of an experimental paradigm developed by Bocanegra and Hommel (2014) was used in this study. The paradigm consists of two separate tasks, which were originally termed “automatic” and “control” task. As they differ with respect to the complexity of task rules and thus the required amount of cognitive control, we however, decided to refer to them as “easy” and “hard” task, respectively.

**FIGURE 1:**
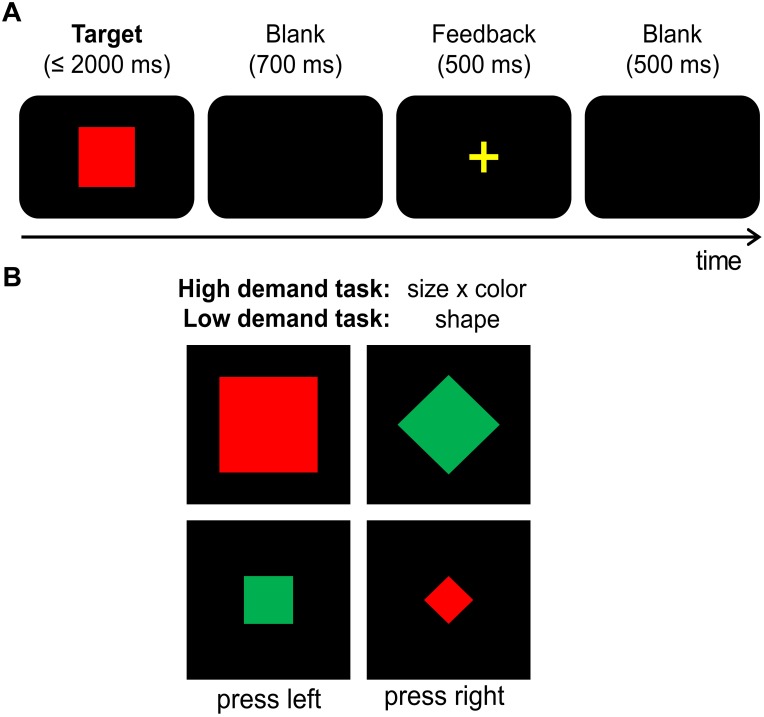
**(A)** Illustration of the task. Each trial started with the target presentation, which was either terminated by the first response or after 2000 ms had elapsed (in this case, the trial was coded as a “miss”). The target was followed by a 700 ms blank screen, a 500 ms feedback (“+” for correct and “–” for incorrect or missed responses) and a second 500 ms blank screen. **(B)** Illustration of employed target stimuli. Targets could vary in shape (square vs. diamond), size (small vs. large) and color (green vs. red). Please note that those are examples and not all of the employed stimulus combinations are illustrated here. The easy task required left button presses for squares and right button presses for diamonds. The hard task required left button presses for targets that were either large and red OR small and green while right button presses were required for targets that were either large and green OR small and red. Hence, the two exemplary stimuli on the left of this graph required a left hand response while the two stimuli on the right on the graph required a right hand response in both of the tasks.

According to the protocol adapted from Bocanegra and Hommel (2014) and to keep potential order effects constant, all participants first performed the hard task and then the easy task while seated at a distance of 57 cm from a 17″ monitor. “Presentation” software (version 14.9. by Neurobehavioral Systems, Inc.) was used for stimulus presentation, response recording, and EEG triggers. Each trial started with the presentation of a single visual stimulus in the center of a black screen for 2000 ms, or until a response was given (cf. **Figure 1A**). Stimuli varied in shape (square or diamond), color (green or red) and size (small/∼2.5 cm diameter or large/∼5 cm diameter). All combinations of stimulus features were presented equally often in both tasks (cf. **Figure 1B**). In the easy task, participants were instructed to respond only to the shape of the stimuli: Whenever the target stimulus was a diamond, the left Ctrl button of a regular keyboard had to be pressed with the left index finger, and the right Ctrl button had to be pressed with the right index finger when the target stimulus was a square. For the hard task (originally termed “control task” by Bocanegra and Hommel, 2014), participants were asked to respond to a combination of size and color of the target stimulus (cf. **Figure 1B**): Whenever the target stimulus was large and red or small and green, the left Ctrl button had to be pressed. When the target stimulus was large and green or small and red, participants had to press the right Ctrl button. In case of no response was given within 2000 ms, stimulus presentation was terminated and the trial was coded as a “miss.” 700 ms after target stimulus offset, a 500 ms feedback was given to inform the participants whether their response was correct (“+”) or incorrect (“–”). This was followed by a fixation cross for 500 ms before the next trial was presented. Each task was subdivided into 5 equally sized blocks with a total of 480 trials. Behavioral measures (accuracy and the mean response times of correct responses) were separately collected for the low and hard tasks.

### Formation of Cognitive Control Adaptability Groups

In order to examine *how* individuals differently adapt to varying levels of cognitive control induced by task rule complexity manipulation, individuals were classified into a “low” and “high” adaptability groups based on their task performance. Two equally large groups were formed by means of a median split of a performance score. For this performance measure, we employed the following equation, which takes speed-accuracy tradeoff effects into account:

$$\text{Adaptability Score=}\frac{{ACC}_{easy}}{{RT}_{easy}}-\frac{{ACC}_{hard}}{{RT}_{hard}}$$

Here, *Acc* is the accuracy in percent and *RT* is the hit RT in ms. When dividing *Acc* by *RT*, (relatively) larger outcomes indicate a better performance, which is characterized by faster and/or more correct responses. By subtracting the performance score of the hard task from that of the easy task, the adaptability score (Easy task: min = 0.14, max: 0.28, range = 0.14, *SD* = 0.028; Hard task: min = 0.08, max: 0.20, range = 0.12, *SD* = 0.021) reflects the magnitude of behavioral performance differences between the hard and easy task: Individuals with large adaptability scores show larger performance differences between tasks than individuals with small adaptability scores. Based on the assumption that differences in adaptability should be mainly driven by performance differences in the easy task (see behavioral results section for confirmation), we named the two groups resulting from a median split of the adaptability score “high adaptability group” and “low adaptability group.” While it has been shown that a median split lowers experimental power and increases the risk of type I errors (Wicherts and Scholten, 2013), it is important to consider that a binary classification is a mandatory requirement for our machine learning approach (Kleinbaum et al., 2013). Machine learning algorithms require a strict and objective criterion for classification (Kleinbaum et al., 2013); e.g., to classify individuals as good or bad performers: Other than research on topics concerning a strictly categorical distinction, psychological research on inter-individual performance differences can usually not provide fixed or objective cutoffs for classifying human behavior. The reason for this is that behavioral performance can only be judged as “good” or “bad” in relation to the performance of others. Thus, performance rating always depends on what comparable individuals are capable of and behavioral parameters of any subject are compared to the behavior observed in either large groups (typically 50 to several hundred individuals) or two or more different experimental groups are contrasted.

### EEG Recording and Data Processing

EEG recordings were made using 60 sintered Ag/AgCl ring electrodes located at standard equidistant scalp positions with a sampling rate of 500 Hz, using electrode Fpz as reference (customized BrainCap Fast‘n Easy sub-inion model EEG caps). All electrodes were connected to a QuickAmp amplifier (BrainProducts Inc.) and impedances were kept below 5 kΩ. After recording with the Brain Vision Recorder (Brain Products Inc.), the data were average-referenced, down-sampled and interpolated to 256 Hz and a band-pass filter (IIR filter from 0.5 Hz to 20 Hz at a slope of 48 db/oct) was applied using Brain Vision Analyzer (Brain Products Inc.). Subsequently, a raw data inspection was conducted in order to manually remove pauses and rare technical artifacts. For removing periodically recurring artifacts including eye -movements, blinks and ECG artifacts, an independent component analysis (ICA; Infomax algorithm) was applied. Segments of trials with correct responses were separately formed for all experimental conditions. Stimulus-locked segments ranged from -2000 ms before to 2000 ms after the onset of the target stimulus and response-locked segments 2000 ms around the response. The segments underwent an automatic artifact rejection (rejection criteria allowed for a maximal value difference of 200 μV in a 200 ms interval and excluded activity below 0.5 μV in a 100 ms interval). Subsequently, a current source density (CSD) transformation was run to obtain a reference-free evaluation of the electrophysiological data (Perrin et al., 1989) with the order of splines set to 4 and the maximal degree of legendre polynomials set to 10. The CSD transformation furthermore serves as a spatial filter that helps to identify electrodes that best reflect activity related to cognitive processes as it accentuates the scalp topography (Nunez and Pilgreen, 1991; Tenke and Kayser, 2012; Kayser and Tenke, 2015). For the stimulus-locked segments a baseline correction was applied to the interval from -300 ms to 0 ms before stimulus onset. For the response-locked segments, a baseline correction was applied to the interval from 300 to 400 ms after the response. Segments were then separately averaged for each participant and condition. Next, different ERP components were quantified at the single-subject level. Electrodes for ERP quantification were chosen on the basis of a visual inspection of the scalp topography, which was validated and confirmed by a procedure described in (Mückschel et al., 2013): For each ERP component, a search interval was defined, in which the respective component was expected to be maximal. Then, the mean amplitude within each of these search intervals was extracted for all 60 electrodes. Each electrode was then compared to the average of all other electrodes using Bonferroni-correction for multiple comparisons. Only electrodes that showed significantly different mean amplitudes than the average were chosen. Of note, this procedure revealed the same electrodes as previously chosen on the basis of visual inspection of the scalp topography plots. The stimulus-locked P1 ERP amplitudes at electrodes P7 and P8 were quantified by extracting the average voltage in the time window ranging from 95 to 105 ms. The stimulus-locked N1 ERP amplitudes at electrodes P7, P8, P9, and P10 were quantified by extracting the average voltage in the time window ranging from 170 to 180 ms. The stimulus-locked N2 and fronto-central P3 amplitudes were quantified at electrode Cz in the time window ranging from 230 to 250 ms and 500 to 700 ms respectively. The stimulus-locked N450 amplitudes were also quantified at electrode Cz. Due to a latency shift between the two tasks, amplitudes were quantified for the easy task in the time window ranging from 380 to 400 ms, and in the hard task in the time window ranging from 395 to 415 ms. The stimulus-locked parietal P3 amplitude was quantified at electrodes PO1 and PO2 in the time window from 305 to 325 ms. The response-locked parietal P3 amplitude was quantified at electrode Pz in the time window from -35 to -25 ms.

### sLORETA Analyses

To examine what functional neuroanatomical networks are modulated during high and low cognitive control demands and how they are modulated between the two formed groups, we conducted source localization using sLORETA (standardized low resolution brain electromagnetic tomography; Pascual-Marqui, 2002) from the LORETA-Key software program (Pascual-Marqui, 2002), which provides a unique solution to the inverse problem (Pascual-Marqui, 2002; Marco-Pallarés et al., 2005). For sLORETA, the intracerebral volume is partitioned into 6239 voxels at 5 mm spatial resolution. Then, the standardized current density at each voxel is calculated in a realistic head based on the MNI152 template (Mazziotta et al., 2001). sLORETA provides reliable results without a localization bias (Sekihara et al., 2005). Moreover, there is evidence from EEG/fMRI and neuronavigated EEG/TMS studies underlining the validity of the sources estimated using sLORETA (Hoffmann et al., 2014; Dippel and Beste, 2015). The voxel-based sLORETA images were compared between the two groups (high adaptability group vs. low adaptability group) in the experimental conditions (hard vs. easy task) using the sLORETA-built-in voxel-wise randomization tests with 2000 permutations, based on statistical non-parametric mapping (SnPM). Voxels with significant differences (*p* < 0.01, corrected for multiple comparisons) between contrasted conditions and groups were located in the MNI-brain.

### Data-Driven Feature Extraction Procedure and Support Vector Machine (SVM) Analysis

Based on a median split of our adaptability score (cf. *Formation of Cognitive Control Adaptability Groups*), a machine learning approach was employed to predict group membership on the basis of the behavioral data from trials where the correct response was executed.

As possible features each of the CSD-transformed data points of each of the 60 EEG channels were extracted with the resolution of the sampling frequency (256 Hz) for every subject. Opposed to the stimulus-locked data, only the response-locked data showed significant main or interaction effects of the group factor. Therefore, all time points were extracted from 300 ms before the onset of the response to 1000 ms after the response for the response-locked averaged segments. All features were normalized into a *z*-score, which was done for two reasons: Firstly, as the z transformation makes all features have a mean of zero and a standard deviation equal to one (Raschka, 2015); the problem of features biasing the feature detection algorithm in case they have different value ranges can be avoided. Secondly, the convergence speed of feature detection algorithms can be increased (Theodoridis and Koutroumbas, 1999).

After the feature normalizing procedure, a feature selection procedure was applied. This is a crucial step for machine learning algorithms, as it eliminates irrelevant features and reduces the problem of having a ‘small’ data set relative to the size of the possible feature set. Both of these factors can otherwise reduce the classifier performance. For the feature selection procedure, an optimal subset of features is selected from the original feature set. Feature selection algorithms can be classified as either using “filter” or “wrapper” methods (Guyon and Elisseeff, 2003). Independent of the chosen classifier the filter method selects a subset of features according to general data characteristics, whereas the wrapper methods require a predetermined classifier and evaluate features according to their performance to discriminate between classes (Guyon and Elisseeff, 2003). Wrapper methods usually lead to better results than filter methods (Saeys et al., 2007), but are significantly slower. One way to avoid this problem is to combine filter and wrapper methods. Therefore, filter methods are applied first to select some features, which are then used as input for wrapper methods. This was done in the current study with *t*-test and sequential floating forward selection (SFFS) methods (Saeys et al., 2007). For that, a *t*-test is calculated between the two groups using the median split procedure for each time point (i.e., feature). If the *p*-value is below 0.01, this time point (feature) is selected. Then these selected features are used as input for the SFFS algorithm. The SFFS method combines a sequential forward selection (SFS) and sequential backward selection (SBS) algorithm (Chandrashekar and Sahin, 2014; Khazaee et al., 2016). The SFS starts from an empty set of features and sequentially adds features that result in the highest classifier accuracy when being combined with the features that have already been selected. The SBS works in the opposite direction. In SFFS, each feature selection step comprises SFS and SBF (Chandrashekar and Sahin, 2014; Khazaee et al., 2016) and were implemented in MATLAB 2017a (Mathworks Inc.). Then, the selected features are fed to a support vector machine (SVM) employing a radial basis function (RBF) kernel, using MATLAB 2017a (Mathworks Inc.) and the LIBSVM toolbox.

Support vector machines are supervised learning algorithms that project input data into high-dimensional feature space to determine a hyper-plane which is able to optimally separate the groups. Since neuroimaging studies usually deal with a ‘small’ number of subjects, the result of the SVM method was cross-validated in this study. For this, the k-fold cross-validation (Arlot and Celisse, 2010) method was used, as the high variance of the classification accuracy and the computation time are two major problems of the Leave One Out Cross Validation (LOOCV) method (Lee and Verri, 2002). The k-fold method randomly divides the data into k portions in which k-1 portion is considered as training data and other as testing data. By continuing this k-times, all subjects in the data set are part of the testing and training set. The resulting classification accuracy is the average of the all k-folds (Arlot and Celisse, 2010). Usually, the value of k is between 5 to 10 in machine learning. We used *k* = 10 in this study. This means that for each extracted feature there were 10 estimations of the predictability of behavioral performance. Using the data from the *k* = 10 estimations, we calculated the 99% confidence bounds for each feature. These confidence bounds were then used to examine (i) in how far each feature provides a significant increase in the predictability of group membership on the basis of the behavioral data. If the difference between the features is significant, this is indicated by no overlap between the calculated 99% confidence bounds. Of note, the applied machine learning approach minimizes the risk of ending up with false positive features: Whereas there is still a risk for false positive features to survive feature selection and enter the machine learning approach, the subsequent k-fold validation procedure minimizes the risk of false positives being ultimately selected as a predictive feature as it mixes and recombines the sample many times, thus strongly decreasing the likelihood of false positives having a strong and consistent effect. In order to see that selected features were not selected by chance, we ran permutation tests. The data were randomly divided into two groups and SVM was employed for prediction the group membership. The random division as well as using SVM was continued 1000 times. Finally, the percentage of how many times (out of 1000) the selected feature from the original data (with true label) has higher accuracy than randomly assigned group label was calculated.

### Statistical Analysis

Behavioral and electrophysiological data were analyzed using separate mixed-effects ANOVAs using the within-subject factor “task” (low vs. high demand) and the between-subjects factor “group” (high vs. low adaptability group). Greenhouse–Geisser corrections were applied when necessary. *Post hoc* tests were Bonferroni-corrected whenever necessary. Linear regression analyses were run separately for each ERP with the adaptability score. As not all of the values were normally distributed in both groups, we conducted additional non-parametric *post hoc* tests (Mann–Whitney *U* tests) whenever necessary. In the results section, the reported mean values are followed by the standard error of the mean (SEM) as a measure of variance.

## Results

### Behavioral Data

**FIGURE 2:**
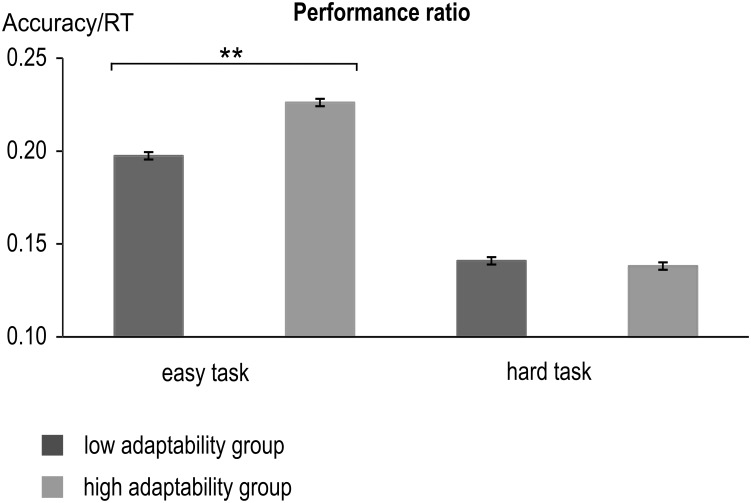
Performance ratio (Accuracy divided by RT) of the low and high adaptability groups for the low and hard tasks. Error bars indicate standard errors (^∗∗^*p* < 0.001). Both groups showed a larger performance ratio (i.e., better performance) in the easy task than in the hard task. In the easy task, performance ratio was furthermore larger for the high adaptability group than for the low adaptability group.

The behavioral data are illustrated in **Figure 2**. The analyses for RT and accuracy can be found in the **Supplementary Material**.

The analysis of the performance ratio (i.e., accuracy divided by hit RT) revealed a main effect of task [*F*(1,225) = 8179.99; *p* < 0.001; $\eta_{p}^{2}$ = 0.973], with better performance in the easy task (0.212 ± 0.002) than in the hard task (0.14 ± 0.001). When we ranked-order Ps by overall adaptability, there was indeed a difference between the high and low adaptability group and ‘high’ performers, [*F*(1,225) = 19.47; *p* < 0.001; $\eta_{p}^{2}$ = 0.08], with better overall performance in the high adaptability group (0.182 ± 0.002) than in the low adaptability group (0.169 ± 0.002). Lastly, there was an interaction of task^∗^group [*F*(1,225) = 391.81; *p* < 0.001; $\eta_{p}^{2}$ = 0.635]. Subsequent *post hoc t*-tests showed that performance differed only in the easy task [*t*(225) = -8.87, *p* < 0.001], where high adaptability group (0.226 ± 0.023) performed better than the low adaptability group (0.0197 ± 0.026). No such difference could be found in the hard task [*t*(225) = 1.10, *p* = 0.299]. As not all of the values were normally distributed in both groups, we additionally conducted non-parametric post-hoc tests (Mann–Whitney *U* tests). They confirmed the findings of the *post hoc t*-tests and also showed significant group differences for the easy task (*p* < 0.001), but not for the hard task condition (*p* = 0.368). Consequentially, the difference between the easy task and the hard task was larger for the high adaptability group (0.088 ± 0.011) than for the low adaptability group [0.057 ± 0.013; *t*(225) = -19.79, *p* < 0.001]. This was also confirmed by a non-parametric *post hoc* test (Mann–Whitney *U* tests), which showed the performance ratio differences between hard and easy task to differ significantly between the groups.

In sum, the behavioral analysis showed that the median split had produced two significantly different groups, as reflected by the interaction of task and group.

### Electrophysiological Data

The participants were divided into high and low performers based on their behavioral data and applying the adaptability score formula (see *Formation of Cognitive Control Adaptability Groups*). In a next step, the EEG data was analyzed to determine the underlying processing contributing to differences in the adaptability toward varying cognitive control demand. According to the literature, there are several potential contributors. The results of the analyses are reported here according to their occurrence along the action cascade.

### The Role of Early Visual Attention (P1 and N1)

**FIGURE 3:**
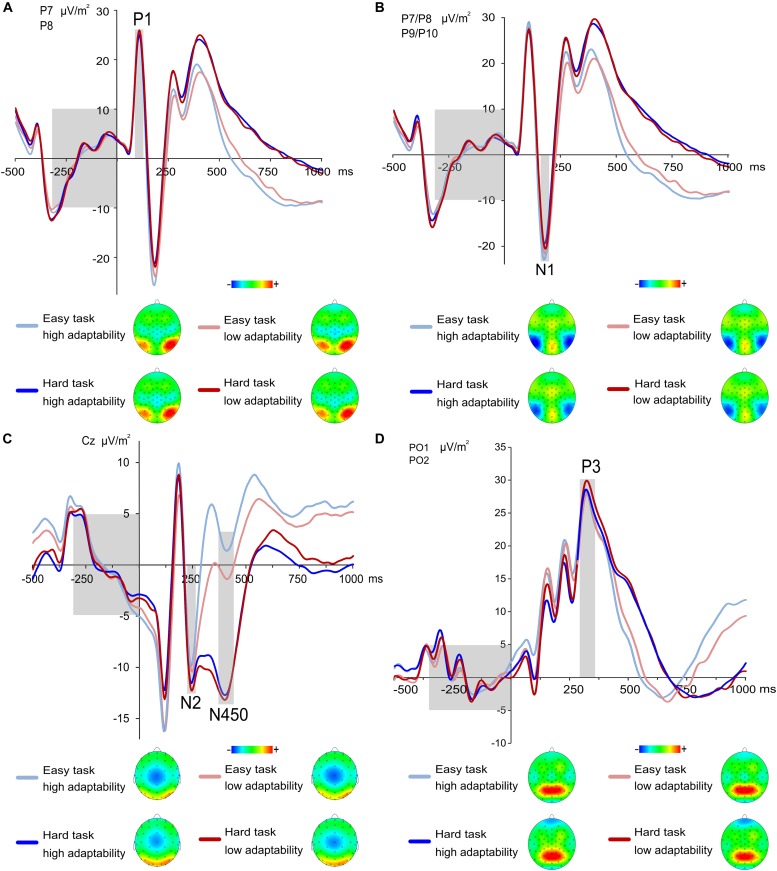
**(A)** Grand means and topographic plots of the P1 at electrode P7 and P8. Time point zero denotes the time point of the target stimulus onset, the light gray boxes illustrate the ERP baseline from –300 to 0 ms (left) and time range each effect is averaged across (right). ERPs of the low adaptability group are denoted by red color, while ERPs of the high adaptability group are denoted by blue color. The easy task is denoted in a lighter shade of the respective colors than the hard task. For the P1, amplitudes of the hard task were larger compared to easy task. **(B)** Grand means of the N1 at electrode P7–P10. Time point zero denotes the time point of the target stimulus onset, the light grey boxes illustrate the ERP baseline from –300 to 0 ms (left) and time range each effect is averaged across (right). ERPs of the low adaptability group are denoted by red color, while ERPs of the high adaptability group are denoted by blue color. The easy task is denoted in a lighter shade of the respective colors than the hard task. For the N1, amplitudes of the easy task were larger compared to hard task. **(C)** Grand means of the stimulus-locked N2 and N450 at electrode Cz. The topographic plots are displayed for the N450, as the main effect of task was only visible in the topographic plots in the N450 time window. Time point zero denotes the time point of the target stimulus onset; the light gray boxes illustrate the ERP baseline from –300 to 0 ms (left) and time range the N2 effect (middle) and N450 effect (right) is averaged across. ERPs of the low adaptability group are denoted by red color, while ERPs of the high adaptability group are denoted by blue color. The easy task is denoted in a lighter shade of the respective colors than the hard task. **(D)** Grand means and topographic plots of the stimulus-locked P3 at electrode PO1 and PO2. Time point zero denotes the time point of the target stimulus onset, the light gray boxes illustrate the ERP baseline from –300 to 0 ms (left) and time range each effect is averaged across (right). ERPs of the low adaptability group are denoted by red color, while ERPs of the high adaptability group are denoted by blue color. The easy task is denoted in a lighter shade of the respective colors than the hard task.

The P1 and N1 ERP are shown in **Figures 3A,B**, respectively. The analysis of P1 amplitudes showed a significant main effect of task [*F*(1,225) = 10.83; *p* < 0.001; $\eta_{p}^{2}$ = 0.046] with larger amplitudes in the easy task (27.9 μV/m^2^ ± 1.23) than in the hard task (26.72 μV/m^2^ ± 1.25). All other effects were non-significant (all *F* ≤ 2.64; all *p* ≥ 0.106). A linear regression was run between adaptability score and P1 amplitudes. A linear regression between adaptability score and P1 amplitudes showed no linear relationship for the P1 amplitudes of low and hard task and the adaptability score [easy task: *F*(1,225) = 0.017, *p* = 0.896, hard task: *F*(1,225) = 0.258, *p* = 0.612].

The analysis of N1 amplitudes revealed a significant main effect of task [*F*(1,225) = 66.64; *p* < 0.001; $\eta_{p}^{2}$ = 0.229] with larger amplitudes in the easy task (-25.03 μV/m^2^ ± 1.34) than in the hard task (-21.56 μV/m^2^ ± 1.28). All other effects were non-significant (all *F* ≤ 3.46; all *p* ≥ 0.076). As for the N1 amplitudes, a linear regression between adaptability score and N1 amplitudes showed that the N1 amplitudes could not significantly predict the adaptability score [easy task: *F*(1,225) = 0.180, *p* = 0.672, hard task: *F*(1,225) = 0.168, *p* = 0.682].

### The Role of Conflict (N2 and N450)

The stimulus-locked N2 and N450 are shown in **Figure 3C**. The analysis of the N2 amplitudes yielded a significant main effect of task [*F*(1,225) = 12.23; *p* = 0.001; $\eta_{p}^{2}$ = 0.055] with larger amplitudes in the easy task (-9.19 μV/m^2^ ± 0.95) than in the hard task (-11.15 μV/m^2^ ± 0.95). All other effects were non-significant (all *F* ≤ 0.44; all *p* ≥ 0.506). Moreover, a linear regression was performed between adaptability score and N2 amplitudes. The analysis revealed that the N2 amplitudes could neither significantly predict the adaptability score for easy task [*F*(1,225) = 0.398, *p* = 0.529] nor for the hard task, [*F*(1,225) = 0.209, *p* = 0.648].

The analysis of the N450 amplitudes yielded a significant main effect of task [*F*(1,225) = 337.18; *p* < 0.001; $\eta_{p}^{2}$ = 0.600] with larger amplitudes in the easy task (0.7 μV/m^2^ ± 0.88) than in the hard task (-12.51 μV/m^2^ ± 0.8). All other effects were non-significant (all *F* ≤ 1.99; all *p* ≥ 0.16). Of note, the finding of larger task differences in the N450 than in the N2 is well in line with the results we observed in a previous publication using a variation of the task used for the current paper (Stock et al., 2016c). Furthermore, a linear regression between adaptability score and N450 amplitudes showed that the N450 amplitudes significantly predicted the adaptability score in the easy task [*F*(1,225) = 8.590, *p* = 0.004], which however, accounted for only 3.2% of the explained variability in the N450 amplitudes. Yet, for the hard task, the linear regression showed no significant prediction of the adaptability score [*F*(1,225) = 0.494, *p* = 0.483].

#### The Role of Response-Selection Processes (P3)

The stimulus-locked fronto-central P3 is shown in the **Supplemental Material**. The analysis of the fronto-central P3 revealed a main effect of task [*F*(1,225) = 76.078, *p* < 0.001; $\eta_{p}^{2}$ = 0.271] with higher amplitudes in the easy task (6.19 μV/m^2^ ± 0.64) compared to the hard task (1.93 μV/m^2^ ± 0.66). No other main or interaction effects were significant (all *F* ≤ 2.907; *p* ≥ 0.090). A linear regression between adaptability score and stimulus-locked fronto-central P3 amplitudes also showed that the parietal P3 amplitudes did not significantly predict the adaptability score in the easy task [*F*(1,225) = 0.787, *p* = 0.376] or in the hard task [*F*(1,225) = 0.230, *p* = 0.632].

**FIGURE 4:**
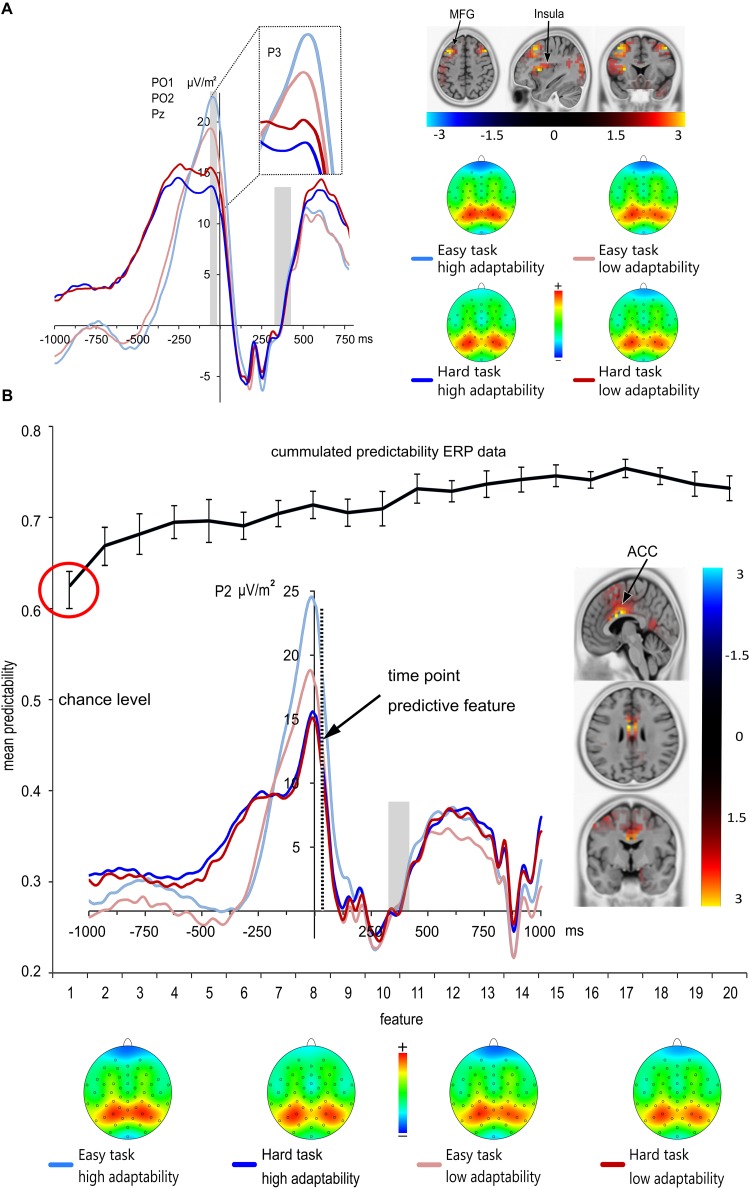
**(A)** Grand means and topographic plots of the response-locked P3 averaged at electrodes PO1, PO2 and Pz. Time point zero denotes the time point of the response. The baseline interval was set from 300 to 400 ms post response; the light gray boxes illustrate the ERP baseline from –300 to 0 ms (right) and time range each effect is averaged across (left). ERPs of the low adaptability group are denoted by red color, while ERPs of the high adaptability group are denoted by blue color. The easy task is denoted in a lighter shade of the respective colors than the hard task. Response-locked P3 amplitudes were significantly larger in the easy task than in the hard task. Additionally, the difference between hard and easy task was larger in the high adaptability group than in the low adaptability group. sLORETA analyses revealed that the size differences in the task effect across groups [operationalized by the contrast of High adaptability group (easy task –hard task) – Low adaptability group (easy task –hard task)], was based on activation differences in the middle frontal gyrus (MFG) (BA9), the insula (BA13) and the cuneus (BA 18). The sLORETA color bar denotes the critical *t*-values. **(B)** Results from the classification analysis using the time domain ERP data. The mean predictability is given depending on the number of features. The black curve in the figure shows the cumulative mean predictability. The error bars represent the 99% confidence level bounds. As indicated by the red circle, the confidence bounds were not overlapping for the first and the second feature. The ERP curve of the first predictive feature is shown. Here, the dashed vertical line in the response-locked ERP plot denotes the exact time point (35 ms after the response) of the first feature at electrode P2. sLORETA analyses revealed that the size differences in the task effect across groups [operationalized by the contrast of High adaptability group (easy task –hard task) – Low adaptability group (easy task –hard task)] at the time point of the predictive feature was due to activation differences in the anterior cingulate gyrus (BA 24; BA 33). The sLORETA color bar denotes the critical *t*-values. ERPs of the low adaptability group are denoted by red color, while ERPs of the high adaptability group are denoted by blue color. The easy task is denoted in a lighter shade of the respective colors than the hard task.

The stimulus-locked parietal P3 is shown in **Figure 3D**. The analysis of the stimulus-locked parietal P3 amplitudes showed no significant main or interaction effects (all *F* ≤ 1.946; *p* ≥ 0.164). Moreover, the linear regression run between adaptability score and stimulus-locked parietal P3 amplitudes also showed that stimulus-locked parietal P3 amplitudes did not significantly predict the adaptability score in the easy task [*F*(1,225) = 0.006, *p* = 0.938] or in the hard task [*F*(1,225) = 0.649, *p* = 0.421]. The response-locked P3 ERP is shown in **Figure 4A**.

The analysis of the response-locked parietal P3 amplitudes revealed a significant main effect of task [*F*(1,225) = 76.75; *p* < 0.001; $\eta_{p}^{2}$ = 0.254] with larger amplitudes in the easy task (21.04 μV/m^2^ ± 0.95) than in the hard task (14.66 μV/m^2^ ± 0.85). There was no main effect of group [*F*(1,225) = 0.06; *p* = 0.804], but an interaction of task^∗^group [*F*(1,225) = 6.41; *p* = 0.012; $\eta_{p}^{2}$ = 0.028]. Subsequent post-hoc t-tests revealed that both groups have larger amplitudes in the easy task than in the hard task [low adaptability group: low demand 19.91 μV/m^2^ ± 1.24, high demand 15.38 μV/m^2^ ± 1.17; *t*(112) = 4.6, *p* < 0.001; high adaptability group: low demand 22.17 μV/m^2^ ± 1.44; high demand 13.94 μV/m^2^ ± 1.23; *t*(112) = 7.69, *p* < 0.001]. Yet, the difference between hard and easy task was larger in the high adaptability group (8.23 μV/m^2^ ± 1.07) than in the low adaptability group [4.54 μV/m^2^ ± 0.99; *t*(225) = 2.53; *p* = 0.012]. Additionally, a non-parametric *post hoc* test (Mann–Whitney *U* test) was performed, as not all of the values were normally distributed in both groups. This confirmed the findings of the *post hoc t*-tests and showed that the amplitude difference between the hard and easy task was larger for the high adaptability group than for the low adaptability group (*p* = 0.033). A linear regression was run between the adaptability score and the response-locked parietal P3 amplitudes. It revealed no statistically predictive value of the response-locked P3 amplitudes on the adaptability score for the easy task [*F*(1,225) = 2.276, *p* = 0.133] and the hard task [*F*(1,225) = 1.743, *p* = 0.188].

Using sLORETA (sLORETA maps were corrected for multiple comparisons using randomization test based on statistical non-parametric mapping, SnPM), we examined which functional neuroanatomical sources were associated with the task^∗^group interaction during the time window where the response-locked P3 amplitude was quantified (-35 ms to -25 ms before the response). The sLORETA analyses comparing the task differences between both groups suggested that this effect was associated with larger activation differences between tasks in the cuneus (BA 18), the insula (BA 13) and in the middle frontal gyrus (BA 9) for the high adaptability group than for the low adaptability group.

In short, none of the ERP amplitudes, which had been quantified in a stimulus-locked fashion (i.e., P1, N1, N2, and P3) showed significant main or interaction effects of the group factor (i.e., a group^∗^task interaction; all *F* ≤ 3.46; *p* ≥ 0.076). As expected, the N450 showed a descriptive trend toward slightly smaller amplitudes in the low demand condition of the high adaptability group as compared to the in the low demand condition of the low adaptability group. Yet, this effect failed to reach significance. To substantiate the assumption that none of the stimulus-locked ERP amplitudes showed significant main or interaction effects of the group factor, Bayesian analyses were conducted as suggested by Wagenmakers (2007) using the template by Masson (2011). These analyses yield the value of p_BIC_(H1| D), which is the probability of the alternative hypothesis being true, based on the obtained data. According to Raftery (1995), values below 0.5 are in favor of the null hypothesis (i.e., indicate that the null hypothesis is more likely to be true than the alternative hypothesis), values between 0.5 and 0.75 are interpreted as weak evidence, values between 0.75 and 0.95 are interpreted as positive evidence, values between 0.95 and 0.99 are interpreted as strong evidence, and values above 0.99 are interpreted as very strong evidence in favor of the alternative hypothesis. For the interaction group ^∗^ task we obtained a p_BIC_ (H1| D) = 0.199 for the P1 and a p_BIC_ (H1| D) = 0.245 for the N1. For the N2 the p_BIC_ (H1| D) = 0.078 was found and for the N450 the p_BIC_ (H1| D) = 0.152. Finally, a p_BIC_ (H1| D) = 0.149 was found for the stimulus-locked P3. Altogether, these results provide strong and reliable evidence for the rejection of the alternative hypothesis/are clearly in favor of the null hypothesis. Yet, only the response-locked P3 nicely reflected both task demand and group differences as observed on the behavioral level. Specifically, the task demand-induced amplitude difference (low > high) was larger in the group that also showed larger behavioral task performance differences.

### Machine Learning Analysis

The results of the SVM analysis of our ERP data are shown in **Figure 4B**. As outlined in the methods section, we used the k-fold method (*k* = 10) to evaluate the predictability of behavioral performance using CSD transformed ERP data. This means that for each extracted feature there were 10 varying estimations of the predictability of behavioral performance. Using the data from the *k* = 10 estimations we calculated the 99% confidence bounds for each feature. A significant difference is indicated by no overlap between the calculated 99% confidence bounds.

As different modulations in the adaptability toward varying cognitive control demands were not found to be reflected in modulations of stimulus-locked ERP data and these adaptability differences were mainly driven by differences in the easy task, we decided to only analyze and report machine learning results from response-locked ERP data in the easy task (**Table 1**). It may be argued that it is inconsistent to restrict a data-driven approach to detect new, possibly relevant neurophysiological features as outlined in the introduction. However, it is important to consider that there is always a risk of false positive results.

**Table 1:** Summary of the extracted features showing feature number, electrode site, time point in ms of the extracted feature after the response was made, the mean predictability and the significance as provided from the *t*-tests used as a filter method in the feature selection step and the corresponding *p*-value for permutation test.

Feature number	Electrode	Time point (ms)	Mean predictability (%)	*p*-value	% in which the prediction is better than all randomly assigned labels
1	P2	35	62.52	0.006	100
2	FC3	797	66.89	0.003	100
3	TP8	320	68.17	0.002	100
4	F5	457	69.48	0.003	100
5	P2	20	69.61	0.003	100
6	C4	996	69.08	0.009	100
7	C5	-277	70.42	0.006	100
8	C4	922	71.37	0.010	100
9	C5	-281	70.52	0.006	100
10	F5	402	70.96	0.007	100
11	Pz	180	73.15	0.009	100
12	F5	410	72.87	0.009	100
13	C5	-273	73.66	0.008	100
14	Pz	184	74.16	0.008	100
15	C4	1000	74.57	0.007	100
16	FC3	574	74.12	0.010	100
17	PO2	320	75.39	0.008	100
18	CP5	-203	74.53	0.008	100
19	CP4	12	73.66	0.005	100
20	F5	574	73.21	0.003	100


The first and best ERP feature was identified 35 ms after the response at electrode P2. Importantly, the position of the P2 electrode and the and ERP waveform identified as the most predictive feature are very closely related to the response-related P3 component. This first ERP feature led to a prediction accuracy for group classification of ∼62.5%, which is significantly above from chance level (50%) as indicated by the 99% confidence bounds. Moreover, permutation test reveals that this feature was not selected by chance, i.e., the accuracy in all of 1000 permutation tests were lower than real accuracy (62.5%). Adding more features led to a numerical increase in prediction accuracy (cf. **Figure 4B**), but this increase was not relevant, as the 99% confidence bounds of the first feature largely overlapped with the prediction accuracy obtained after adding the second feature. The sLORETA contrast at 35 ms after the response (i.e., the time point of the ERP feature which best predicted performance as defined by group membership), was calculated using a single group zero mean test (i.e., ERP_low demand_ = 0). The sLORETA analysis revealed a main activation in the anterior cingulate gyrus (BA 24; BA 33). Notably, the identified feature at 35 ms after the response is only slightly shifted behind the response-locked P3 amplitude and also the source localization results are very coherent. This validates the findings and makes it very unlikely that the effect found for the response-locked P3 reflects a type 1 error.

## Discussion

The aim of this study was to identify the neural dynamics related to metacontrol, i.e., the ability to strategically adapt to different cognitive control demands (Doya, 2008; Goschke and Bolte, 2014; Hommel, 2015; Hommel and Colzato, 2017; Hommel and Wiers, 2017). To identify how and why the level of adaptation to varying cognitive control demands differs between individuals, we formed two equally large groups on the basis of an “adaptability score.” This score reflected how well an individual could disengage cognitive control in the face of low control demands. In order to identify the underlying neurophysiological processes and neuronal sources of those differences, we combined ERP recordings with source localization and machine learning approaches.

Generally, individuals in the high adaptability group seemed to outperform the low adaptability group, as reflected by an overall higher behavioral performance score. This was however, not based on superior performance in the high control demand task, but instead based on better performance in case of low control demands. While this finding might seem counterintuitive at first, it makes a lot of sense when regarding metacontrol as the ability to allocate control resources in a demand-dependent manner, i.e., to selectively exert control only in situations requiring this strategy (Goschke and Bolte, 2014; Hommel and Wiers, 2017). This is well in line with the action control concept by Hommel and Wiers (2017), who assumed that the metacontrol ability is based on a system that manages to find the right balance in the dynamic interplay between extreme ‘persistence’ and extreme ‘flexibility’ of information processing (Dreisbach and Goschke, 2004; Cools and D’Esposito, 2010). In case of low cognitive control demands (i.e., in the easy task), individuals with better metacontrol abilities exert less ‘flexible’ cognitive control and let more ‘persistent’ stimulus-driven automaticity take over. Hommel and Wiers (2017) further assume that selection criteria, such as energy consumption and efficiency, reflect these possible shortages of currently available cognitive resources.

As already stated in the introduction, behavior would be ill-advised by a system that pursues goals in a top-down manner irrespective of the given control requirements (Hommel and Wiers, 2017). It would be much more beneficial and expedient to adjust cognitive control or response selection processes depending on situational requirements (induced via task rule complexity), because both of those processes are known to be capacity-limited (Meyer and Kieras, 1997; Engle and Kane, 2003; Kane and Engle, 2003; Tombu and Jolicoeur, 2003; Lavie et al., 2004; Marois and Ivanoff, 2005; Sigman and Dehaene, 2008; Engle, 2010; Lavie, 2010; Yildiz et al., 2013; Yildiz and Beste, 2015). A strategic adjustment of these processes to task rule complexity would allow to optimize the use of this limited capacity as an important resource. Furthermore, and even more importantly, a strategic reduction of top–down strategies in situations with low control demands most likely allows the participants to benefit from rather automated behavior and response-selection strategies, which is not only less capacity demanding, but also tends to produce faster and less error-prone responses. As the behavioral data were well in line with this assumption, we proceeded with the analyses of ERPs that are known to reflect the control demand differences of our experimental paradigm (Stock et al., 2016d) and might therefore also differentiate individuals with high and low adaptability/metacontrol.

We had expected to find the behavioral group differences to be reflected by central measures of cognitive control and conflict, i.e., the N2 and/or N450 (Larson et al., 2014). Specifically, we had expected to find smaller (i.e., less negative) N450 amplitudes during the low control demand task in individuals with a high adaptability (i.e., metacontrol abilities) than in individuals with low adaptability. The reasoning behind this hypothesis was that if individuals with higher metacontrol are better able to refrain from engaging top–down strategies in situations with low control requirements, this should be reflected in smaller N450 amplitudes, which had previously already shown to be modulated by variations in control demands as induced by task rule complexity (Stock et al., 2016d). But even though both control ERPs showed the expected larger amplitudes in the high demand condition (Botvinick et al., 2004; Folstein and Van Petten, 2008; Larson et al., 2014; Stock et al., 2016c) and there was slight descriptive tendency toward larger N450 amplitudes in the easy task of the high adaptability group as compared to the low adaptability group (cf. **Figure 3C**), the latter failed to reach significance. This effect may have failed to reach significance due to relatively large intra-individual variability, which could have potentially been increased by the relatively temporal large distance from the locking point. Interestingly, a linear regression established that only in the easy task, the N450 amplitudes predicted the adaptability score, which however, only explained very little variance (4.5%). Yet, add-on Bayesian underpinned the fact that neither basic bottom-up perceptual and attentional selection processes reflected by P1 and N1 (Luck et al., 2000; Herrmann and Knight, 2001), nor conflict monitoring processes reflected by the N2 or N450 (Folstein and Van Petten, 2008; Larson et al., 2014), stimulus-driven frontal attention mechanisms during task processing reflected by fronto-central P3 or stimulus-response mapping processes reflected by the stimulus-locked parietal P3 could explain the behavioral differences observed between our two experimental groups. This was further underlined by multiple linear regression analyses showing that the ability to strategically adapt to different cognitive control demands (i.e., the adaptability score) was not predicted by amplitude modulations in most of the processes mentioned above. This suggests that these stimulus-associated processes are unlikely to reflect meta-control processes.

Instead, the behavioral group differences level were paralleled by the modulation of parietal response-locked P3 ERP amplitudes. Specifically, we found these larger P3 amplitude differences to parallel the larger behavioral task differences observed in the high adaptability group. The parietal P3 component that we quantified has previously been found to appear in both stimulus- and response-locked ERPs (Verleger et al., 2005; Stock et al., 2016d), presumably because it represents an intermediate process between stimulus evaluation and responding in choice reaction tasks (Falkenstein et al., 1994a,b; Verleger et al., 2005). It has furthermore repeatedly been linked to action selection or mapping appropriate responses onto perceived task-relevant stimuli (Verleger et al., 2005, 2015; Twomey et al., 2015; Gohil et al., 2016; Petruo et al., 2016; Stock et al., 2016a, 2017; Gohil et al., 2017). The fact that response-locked P3 reflected the observed behavior, while the stimulus-locked P3 showed no effects at all, suggests that stimulus-associated information processing is unlikely to account for the observed behavioral group differences, although it needs to be mentioned that stimulus- and response-locked ERPs like the P3 are not completely independent measures and that both processes partly overlap (Ouyang et al., 2011, 2015b). Yet, it seems much more likely that later response-selection processes could orchestrate the dynamics in the ability to efficiently adapt to varying cognitive control demands. This interpretation seems to be well in line with previous findings that the parietal P3 component reflects variations in cognitive control demands (Verleger et al., 2005; Twomey et al., 2015; Stock et al., 2016d), with lower demands producing larger P3 amplitudes (Stock et al., 2016d). In dual task situations (Polich, 2007) or during processes of early vision (Schubö et al., 2001), this amplitude increase has repeatedly been suggested to reflect the attentional resources left over by the primary task. Given that we found larger task differences in the high adaptability group, this provides further evidence for our hypotheses that metacontrol is defined by the ability to allocate control resources based on the actual demand for top–down control, rather than always exerting control (Goschke and Bolte, 2014; Hommel and Wiers, 2017). The fact that the high adaptability group had slightly larger response-locked P3 amplitudes in the low demand condition suggests that their attentional and control resources were less strained than those of the low adaptability group, probably because this group applied a rather automatic and more expedient stimulus-response mapping strategy. Likewise, the slightly smaller P3 amplitudes of the high adaptability group in the high demand condition suggest that this group may have invested more control resources and thus suffered a greater decrement in the still available residual control capacities than the low adaptability group. Source localization analysis showed the group differences in P3 task effects were associated with activation differences in the middle frontal gyrus (MFG) (BA9) and the insula (BA13). It has been suggested that the insula serves as an integral hub, which is implicated in a plethora of different functions like reinforcement learning, emotion control, and decision-making (Gogolla, 2017) and plays a critical role in cognitive control (Menon and Uddin, 2010). Even more importantly, both insular cortex and the MFG have been shown to be especially engaged during response selection stages of decision-making (Paulus et al., 2005; Karch et al., 2010). In particular, the insula was shown to be more active when subjects selected another response relative to staying with the same choice made on the previous trial (Karch et al., 2010) and the MFG was associated with voluntary response adaptation (Paulus et al., 2005). Therefore, the current findings and interpretation fits well into the current literature on functions of the insula and the MFG during response selection and control and suggests that those two brain regions might play a key role in the identification of control requirements and the subsequent allocation of control resources.

In addition to those classical ERP analyses, we chose to complement our methodological approach with machine learning. The SVM analysis identified an EEG feature which best classified whether a given participant belonged into the high or low adaptability group with a prediction accuracy of 63% (with a chance level of 50%). Future research using this strategy might consider to apply SVM on temporally decomposed data(Mückschel et al., 2017; Wolff et al., 2017), applying an algorithm that reduces intra-individual variability of the data (Ouyang et al., 2011, 2015a,b; Verleger et al., 2014) and could therefore lead to increased classification accuracies. Importantly, the ERP feature that best predicted the behavioral group membership was identified temporally and topographically close to the peak of the response-locked P3 amplitude in the easy task. There are thus coherent findings from two different analyses performed with the data. In line with a number of studies, we found the response-locked P3 peak as well as the best predictive feature to occur around the moment of responding (Verleger et al., 2005, 2016; Saville et al., 2011; Stock et al., 2016c). It is therefore likely that the identified feature also reflects aspects of response selection processes. Interestingly, source localization at the time point of the predictive P3 feature showed that the anterior cingulate cortex (ACC; BA 24; BA 33) was the largest generator of neuronal activity at this specific time point. The reason why different sources were detected by the standard ERP analysis and the SVM analysis is that the ERP feature detected by the SVM was identified at a later time point than the P3 peaks identified by the ERP analysis. While some theories assume that activation in the ACC is mostly useful for boosting weak processes in order to establish top down control (Paus, 2001; Stuss et al., 2005), a complementary conception by Shenhav et al. (2013, 2016) proposes that the role of the ACC during cognitive control can be understood in terms of a single underlying function: the allocation of control based on the evaluation of the EVC. According to EVC, the ideal cognitive control adaptation strategy would be to attribute high amounts of cognitive control in situations, where high cognitive effort is required and low amounts of cognitive control in situations, which can be better processed in an automated fashion with less cognitive effort. The EVC concept might therefore also explain why activation modulation in the ACC was observed at the time point that was most predictive for differences in metacontrol, i.e., the ability to adapt the allocation of top–down resources to different cognitive control demands. As a central hub for response selection and computation of the EVC, the ACC can probably be better modulated by subjects who were able to efficiently allocate cognitive control (high adaptability group) compared to those who rather struggle to adapt to differences in control requirements (low adaptability group). The ACC modulation, which was associated with the modulation of the P3 ERP, may be interpreted as a response selection process based on the evaluation of the EVC. This integration process can either promote simple, fast, and overlearned actions, which are more automatic, or complex, slow, and novel actions, which are more controlled (Hommel and Wiers, 2017; Beste et al., 2018). Thus, it can be argued that the ability to efficiently integrate the relevant information for the response selection process and to strategically change the ‘style’ of the response selection (Hommel and Wiers, 2017) into more automatic or more controlled mode is associated with group differences in the adaptability toward varying cognitive control demands. Since group differences were mainly driven by performance differences in the easy task, it can furthermore be concluded that individuals who are better at strategically adapting to different cognitive control demands seem to especially benefit from reducing the exerted cognitive control level to enable a stronger automatization of their response selection processes in situations where little cognitive control is needed. This allows the conclusion that inter-individual differences in metacontrol capacities might not necessarily emerge from differences in the maximum of cognitive control that can be invested. Instead, the most expedient type of metacontrol seems to allocate control in a rather frugal way, which leaves more room for expedient and less error prone automatic processing strategies that provide the individual with more available/“free” residual cognitive resources.

## Author Contributions

CB and AK-S designed the experiment, conducted data analysis, and wrote the paper. NZ collected the data, conducted data analysis, and wrote the paper. AV conducted data analysis, and wrote the paper.

## Conflict of Interest Statement

The authors declare that the research was conducted in the absence of any commercial or financial relationships that could be construed as a potential conflict of interest.

## Footnotes

1 Please note that for the purpose of this paper, the term “metacontrol” will be solely used to describe the ability to strategically adapt to different cognitive control demands.
