# Supplementary material for: On the Neurophysiological Mechanisms Underlying the Adaptability to Varying Cognitive Control Demands
Source: Front Hum Neurosci. 2018 Oct 16;12:411. doi: 10.3389/fnhum.2018.00411 (PMC6232768; doi:10.3389/fnhum.2018.00411)
Supplement: Supplementary file 1 [file Data_Sheet_1.docx]

**Supplemental material**

**On the neural mechanisms underlying the adaptability to varying cognitive control demands**

Zink N, Stock AK, Vahid A, Beste C

*Behavioral data*

The analysis of the hit RT revealed a main effect of task (*F*(1,225) = 2533.49; *p* < .001; $\eta_{p}^{2}$ = .918), with higher RTs in the hard task (682 ± 7) compared to the easy task (467 ± 4). Furthermore, there was an task*group interaction (*F*(1,225) = 56.11; *p* < .001; $\eta_{p}^{2}$ = .200). Further post-hoc t test showed that RT only differed between the groups in the easy task, with higher RTs in the low adaptability group (498 ± 6) than the high adaptability group (436 ± 6; *t*(225) = 7.86, *p* < .001), but not in the hard task (*t*(225) = -0.10, *p* = .921). As not all of the values were normally distributed in both groups, we additionally conducted non-parametric post-hoc tests (Mann-Whitney-U tests). They confirmed the findings of the post-hoc t-tests and also showed significant group differences for the easy task (p < .001), but not for the hard task condition (p = .838).

The analysis of the accuracy revealed a main effect of task (*F*(1,225) = 332.02; *p* < .001; $\eta_{p}^{2}$ = .596), with higher accuracy in the easy task (97.10 ± 0.17) compared to the hard task (93.06 ± 0.30). Furthermore, there was an task*group interaction (*F*(1,225) = 28.30; *p* < .001; $\eta_{p}^{2}$ = .112). Further post-hoc t test showed that accuracy differences between high and easy task were larger for the high adaptability group (498 ± 6) compared to the low adaptability group (436 ± 6; *t*(225) = 7.86, *p* < .001). As not all of the values were normally distributed in both groups, additionally conducted non-parametric post-hoc tests confirmed the findings of the post-hoc t-tests and also showed differences in the effect size of the accuracy differences between the tasks (p < .001).

*The stimulus-locked fronto-central P3*


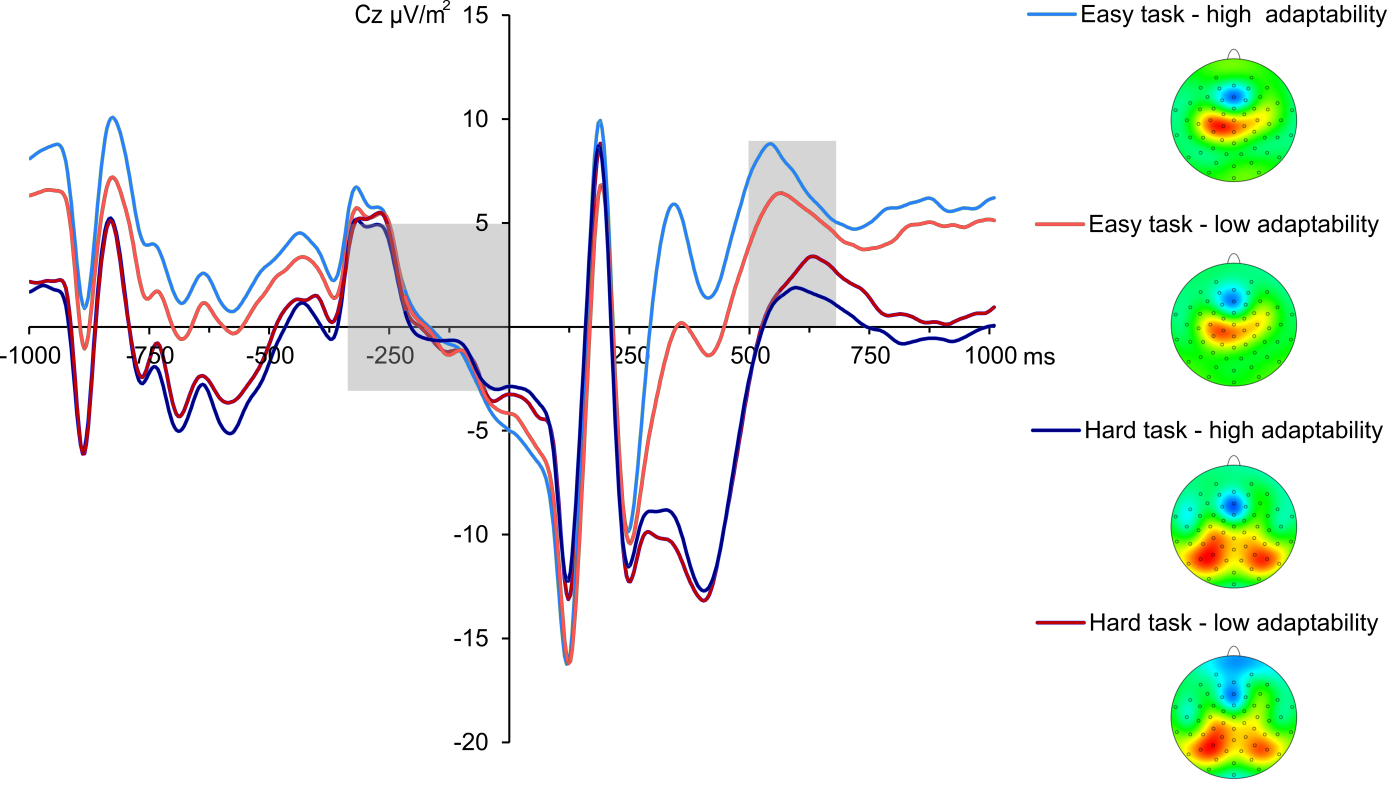


Grand average and topographic plots of the fronto-central P3 at electrode Cz. Time point zero denotes the time point of the target stimulus onset, the light grey boxes illustrate the ERP baseline from -300 to 0 ms (left) and time range each effect is averaged across (right). ERPs of the low adaptability group are denoted by red color, while ERPs of the high adaptability group are denoted by blue color. The easy task is denoted in a lighter shade of the respective colors than the hard task.
